# Alumni Association of the Philadelphia Dental College

**Published:** 1891-10

**Authors:** Alonzo Boice, L. Ashley Faught


### Alumni Association of the Philadelphia Dental College.

At a meeting held April 9th, 1891, all the graduates of the Philadelphia Dental College during the years '86, '87, '88, '89, '90 and '91 were elected members. Those desiring to accept such membership will please send to J. R. C. Ward, D.D.S., Treasurer, 1905 Fairmount Avenue, Philadelphia, their name, address and one dollar entrance fee.

Alonzo Boice, President. L. Ashley Faught, Sec'y.

